# Development of an eLearning intervention for enhancing health professionals’ skills for addressing COVID-19 vaccine hesitancy

**DOI:** 10.3389/fmed.2023.1290288

**Published:** 2023-12-13

**Authors:** Sophia Papadakis, Marilena Anastasaki, Maria Gamaletsou, Xenia Papagiannopoulou, Eftychios Aligizakis, Christos Lionis

**Affiliations:** Clinic of Social and Family Medicine, School of Medicine, University of Crete, Rethymno, Greece

**Keywords:** COVID-19 vaccination, vaccination hesitancy, motivational interviewing, continuing medical education, training, Greece

## Abstract

Like many countries, Greece has faced resistance to coronavirus disease 2019 (COVID-19) vaccination among residents for both the initial and booster doses. Supporting healthcare professionals with delivering brief advice on COVID-19 vaccination may assist with reaching national vaccination targets. We sought to rapidly develop, pilot test, and deploy an eLearning intervention on skills training on effective techniques for addressing COVID-19 vaccine hesitancy for primary health and social care professionals in Greece. A five-part, 1.5-h eLearning was produced in Greek which featured two behavior change techniques, Very Brief Advice (VBA) and Motivational Interviewing (MI) adapted for use in addressing COVID-19 vaccine hesitancy. Six-film-based case studies modeling the use of VBA and MI in the context of challenging scenarios typically seen in Greek health and social settings were produced for the eLearning. The CME was pilot tested using a pre-post design in a small convenience sample (*n* = 17) of health care professionals. Pilot study results found the training provided new knowledge (80%), improved provider skills (80%), and was useful to provider’s clinical practice (90%). There was a mixed effect in provider capability, motivation, and opportunity. Ninety percent of providers strongly agreed or agreed that they planned to use the information and skills provided by the training in their clinical practice. This project has resulted in new training assets for use by health and social professional tailored to the nationally context in Greece including supporting uptake of booster doses of the COVID-19 vaccine.

## Introduction

Key to the success of national coronavirus disease 2019 (COVID-19) vaccination programs is the ability to reach immunization targets for both the initial and booster doses (1).

Vaccine hesitancy among residents may serve to undermine efforts of national governments and has been identified as being a target for both research and intervention (1–4).

Vaccine hesitancy is defined as a delay in acceptance or refusal of vaccines, despite availability of services (2, 3). Willingness to be vaccinated occurs along a continuum with some individuals being ambivalent, while others, strongly refuse to be vaccinated. Vaccine hesitancy can be complex and influenced by the context in which the individual lives and works, personal and family health status and in many cases is not stable (2, 5, 6).

The health care community and, in particular, primary care providers (PCPs) and social care professionals have an important role to play in supporting vaccine uptake in communities which they serve (3, 4, 7–11). Few training resources are available to equip members of the health and social care community on how they can support the COVID-19 vaccine uptake and, in particular, which techniques will increase the likelihood they can influence the behaviors of residents. In order to support PCPs in this role, new training and resources are required to enhance providers’ skill and confidence in addressing COVID-19 vaccine hesitancy among residents (2, 7). Such training needs to be based on existing international best practices but also be locally adapted and disseminated in the local language. In particular, available evidence and practice has identified perceived risk, motivation, and health literacy as important predictors of health-seeking behavior and adherence to COVID-19 measures including vaccination (12).

This brief report summaries recent experience in the development pilot testing of an eLearning intervention on effective techniques for addressing COVID-19 vaccine hesitancy for primary health and social care professionals in Greece.

## Materials and methods

### Design and procedures

**Figure 1:**
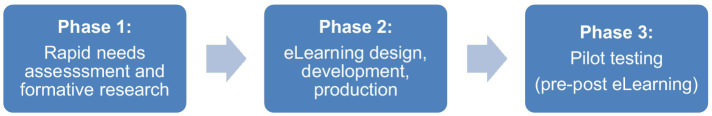
Design.

Figure 1 summarizes the three phases of this project. A rapid needs assessment and formative research was conducted to validate our understanding of vaccine hesitancy and its presentation locally and inform the design of the training (Phase 1). This was followed by a development phase in which the learning objectives, curricula, the adaptation of Very Brief Advice (VBA) and Motivational Interviewing (MI) for addressing COVID-19 vaccine hesitancy, and film -based skills demonstrations were developed (Phase 2). We pilot tested the eLearning and outreach intervention among a sample of PCPs on the island of Crete, Greece (Phase 3).

### Setting and target population and behavior

The target population for the intervention is primary care and social care providers who have contact with patients/persons who report vaccine hesitancy. The target behavior we are seeking to influence is conversations to address COVID-19 vaccine hesitancy with patients in their own clinical practice settings. In the pilot assessment of our training, we included general practitioners/family physicians (licensed or resident) and social care workers practicing in either public or private services. Providers not able to provide informed consent for participation due to any reason were excluded.

### Theoretical framework

The COM-B (‘capability’, ‘opportunity’, ‘motivation’ and ‘behavior’) model, the Health Beliefs Model (HBM) and the Theory of Planned Behavior (TPB) was used to inform the eLearning design and pilot testing design (13–16). Specifically, the intervention targeted the following provider level constructs: provider confidence, attitudes, and motivation, and intentions. Additionally, we considered in the intervention design the influence of cultural factors, local belief systems and risk-communication methods.

### Phase 1 - rapid needs assessment and formative research

The project team conducted a review of existing training assets and knowledge and best practices on very brief advice and motivational interviewing to address COVID-19 vaccine hesitancy. We also conducted a rapid needs assessment with members of the local community in order to validate and enrich understanding of the target audience’s needs. Two semi-structured focus groups were conducted with residents in both rural and urban regions of Crete, Greece. The interviews explored health literacy, perceptions related to COVID-19 and the COVID-19 vaccine, as well as intentions related to COVID 19 and perceived reasoning. Beliefs, barriers, and facilitators for the uptake of COVID-19 vaccination were documented and key themes identified.

### Phase 2 - eLearning intervention design, development, and production

An existing training program published by the World Health Organization was adapted.^1^ The adaptation was informed by focus groups conducted among residents in Crete to identify factors and beliefs associated with ambivalence and hesitancy for COVID-19 vaccination, as well as consultation with a sample of health and social care workers, and expert input. The eLearning intervention provides skills training for PCPs and social care providers in discussing COVID-19 and COVID-19 vaccine with residents and addressing both low confidence in vaccines and indecision, as well as negativity about COVID-19 vaccination. The training program is focussed on: (1) skills training in behavior change techniques including Very Brief Advice (VBA) and Motivational Interviewing (MI) for addressing COVID-19 vaccination hesitancy; (2) patient-centered communication techniques and compassionate care (17–21). Motivational interviewing (MI) is widely used counseling technique for helping people to explore and resolve their uncertainties about changing their behavior (18, 20). It seeks to avoid an aggressive or confrontational approach and steer individuals toward choosing to change their behavior, and to encourage their self-belief.

### Phase 3 - pilot testing

A pre-post pilot evaluation of the CME was completed. Participating PCPs survey at two time points before, immediately following their exposure to the eLearning in order to assess:

a) satisfaction with the eLearning and outreach resources and recommendations for improving the training.
b) changes in capability (confidence), motivation, opportunity, and intentions in addressing COVID-19 vaccine hesitancy with patients.

A convivence sample of family physician and family practice medical residents serving in primary healthcare units and community services in the island of Crete, Greece were invited to participate in the eLearning using official listings from regional healthcare authorities. All providers who agreed to participate in the pilot study provided informed consent and were asked to complete the provider-level surveys immediately before and immediately after the completion of the eLearning. This study was approved by the Research Ethics Committee of the University of Crete (approval number: 121/20.09.2021). In order to reduce respondent bias all data collection occurred via anonymized online survey.

#### Outcome measures

Key demographic characteristics of the pilot study sample were documented. Provider satisfaction with the eLearning was assessed via survey immediately after the training and included the extent to which the training provided useful information, was enjoyable, whether they would recommend the training to colleagues. Free text responses were used to assess what participants enjoyed most about the training and would recommend for improving the training.

We also examined the influence of the intervention on capability, motivation, opportunity, and behavioral intentions at the level of both providers and the population as defined by the COM-B model before and immediately following exposure to the eLearning with responses provided on a five-point Likert scale: (1) strongly agree through to (5) strongly disagree. At the time of this study there were no published tools which adapt assessment of capability, motivation, opportunity and intentions for COVID-19 vaccination and vaccine hesitancy. Our team developed a customized tool for use in this pilot study. We adapted existing tools for the assessment of training programs to the present behavior (16).

*Capability (Confidence):* Providers were asked to rate how confident they felt in raising the issue of COVID-19 vaccination with patients/families/community members using three items.

*Motivation and opportunity*: Four items were used to assess provider motivation to deliver very brief interventions toward COVID-19 vaccination in daily practice and with specific patient populations was assessed. Two items were used to examine provider perception regarding the opportunity to intervene with patients.

*Behavioral intentions:* Intentions were measured as a proxy for clinical practice behaviors. Before and immediately following the eLearning, intentions of providers to deliver very brief interventions toward COVID-19 vaccination were assessed.

#### Data analysis

Descriptive statistics were used to summarize provider demographic data. Non-parametric Wilcoxon Signed Rank tests were used to examine paired differences between timepoints (pre vs. post) as, due to small sample size data were skewed. Test value of *p* were calculated based on the sample of providers for which data was available at both timepoints being compared, as part of providers were lost-to- follow-up (i.e., did not respond to follow-up assessment after three reminding phone calls and/or email contacts). For remaining participants, no missing data were present. Statistical significance of <0.05 was used for all analyses. SPSS was used to analyze the data.

## Results

### Phase 1 - rapid needs assessment and formative research

Six themes were identified as being most pertinent to vaccine hesitancy beliefs locally: (1) concerns about side effects (‘I am worried about the adverse effects of the vaccine, you hear stories in media’), (2) concerns about safety of vaccines (‘I am worried about side effects’), (3) pushback regarding government mandated health-related decision (‘No one can tell me what to do’), (4) beliefs about low risk of susceptibility and illness (‘I am young and healthy and not at risk’), (5) religiosity (‘the church does not believe in vaccination’), and (6) beliefs regarding vaccine efficacy (‘the vaccine does not work, people are who are vaccinated still get infected’).

### Phase 2 - eLearning intervention design, development, and production

A five-part (1.5 h) eLearning was produced. The eLearning features six video vignettes modeling how to assess, communicate and approach common dialogs about COVID-19 and COVID-19 vaccination hesitancy using VBA and MI in the context of challenging scenarios typically seen in Greek health and social settings in regard to COVID-19 vaccination. Modeling behavior change skills has been shown to be an effective technique for increasing PCPs skill and confidence in addressing behavior change with patients and residents (18, 20). Table 1 provides an overview of the training films.

**Table 1:** Skills training films.

Description	Link
George, 65 years, I am worried about adverse reaction to vaccine	https://youtu.be/6tqsXmSJoOU
Eleni, 40 years, side effects of the vaccine	https://youtu.be/xIz_OgAc4wk
Nikos, 30 years, I am young and healthy	https://youtu.be/9y-y7ANycoo
Maria, 75 years, religious beliefs	https://youtu.be/ucNuc6KGWco
Vasillis, 65 years – No one can tell me what to do	https://youtu.be/ynXjAoDHX54
Marina, 60 years – The vaccine does work	https://youtu.be/b7qJY4ExIEc

A digital leaflet on leaflet on COVID-19 Vaccine Hesitancy for Health and Social Care providers was produced which provides a summary of key knowledge and skills related to addressing vaccine hesitancy in health and social settings to reinforce eLearning course content. A graphically designed slide deck for supporting webinar and/or face to face CME delivery was developed to support hybrid learning.

### Phase 3 - pilot testing

A total of 50 out of a list of 200 family physicians practicing in Crete were invited to participate in the pilot study. We also invited 20 family medicine residents affiliated to the university and all providers from 5 social care facilities of Crete. Of all these, 38 providers provided an initial positive response to our invitation, 22 provided informed consent and 17 completed the baseline evaluation. Post-course evaluation was completed by 10 participants. The primary reasons for loss to follow-up was inability to find time to complete eLearning (*n* = 5) and/or lack of interest in the eLearning subject matter (*n* = 2).

Table 2 presents the sociodemographic characteristics of the *N* = 17 healthcare professionals at baseline. Overall, 52.9% of the sample (*n* = 9) were men, with median (IQR) age of 30 (7) years. The majority were resident GPs (*n* = 12 or 70.6%), working in urban areas (*n* = 11 or 67.7%).

**Table 2:** Sociodemographic characteristics and personal vaccination status of healthcare professionals participating in the eLearning pilot evaluation (*N* = 17).

Variable	Value
Gender, *n* (%)	
Male	9 (52.9)
Female	8 (47.1)
Age, median (IQR)	30 (7)
Profession, *n* (%)	
Resident, family medicine	12 (70.6)
General practitioner	4 (23.5)
Other	1 (5.9)
Years of practice, median (IQR)	2 (3)
Setting, *n* (%)	
Rural	6 (35.3)
Urban	11 (64.7)

IQR, interquartile range.

### Provider satisfaction with the training

High levels of satisfaction with the training resources were documented among providers who participated in the pilot study (Table 3). The majority of participating providers agreed or strongly agreed that the training was useful (80%), interesting (90%) and enjoyable (80%). The majority of providers indicated the training provided new knowledge (80%), improved their skills (80%), and was useful to their clinical practice (90%). There was mixed evaluation of the training format and feedback from participants indicated the course duration should be reduced with a focus on applying skills to practice. Eighty percent of participants indicated they would recommend the course to other health care professionals.

**Table 3:** Provider satisfaction with CME and assessment of commercial bias (*N* = 17).

Measure	*n* (%)
Overall, the training was useful	
Strongly agree	1 (10)
Agree	7 (70)
Neutral	2 (20)
Disagree	0 (0)
Strongly disagree	0 (0)
Overall, the training was interesting	
Strongly agree	1 (10)
Agree	8 (80)
Neutral	1 (10)
Disagree	0 (0)
Strongly disagree	0 (0)
Overall, the training was enjoyable	
Strongly agree	1 (10)
Agree	7 (70)
Neutral	2 (20)
Disagree	0 (0)
Strongly disagree	0 (0)
The training improved my skills	
Strongly agree	2 (20)
Agree	7 (70)
Neutral	1 (10)
Disagree	0 (0)
Strongly disagree	0 (0)
I would recommend this training to others	
Strongly agree	2 (20)
Agree	6 (60)
Neutral	1 (10)
Disagree	1 (10)
Strongly disagree	0 (0)
I was satisfied with the online training format	
Strongly agree	2 (20)
Agree	5 (50)
Neutral	2 (20)
Disagree	1 (10)
Strongly disagree	0 (0)
The training provided me with new knowledge	
Strongly agree	2 (20)
Agree	6 (60)
Neutral	2 (20)
Disagree	0 (0)
Strongly disagree	0 (0)
The training was useful to my clinical practice	
Strongly agree	3 (30)
Agree	6 (60)
Neutral	1 (10)
Disagree	0 (0)
Strongly disagree	0 (0)

### Provider capability, motivation, opportunity, and behavioral intentions

As shown in Table 4, there were some positive but non-significant changes in provider confidence in addressing vaccine hesitancy following exposure to the training. A positive effect documented for provider confidence on advising on the benefits of COVID-19 vaccine hesitancy that was not statistically significant (*p* = 0.059).

**Table 4:** Changes in provider capability (confidence), motivation, opportunity and intentions related to COVID-19 vaccine hesitancy pre and post training.

Measure	Median (IQR)		
	Pre-training *n* = 17	Post-training *n* = 10	Value of *p*^d^
Capability (Confidence)^a^ I am confident in….			
Raising the issue of COVID-19 vaccination	4 (1)	4 (0)	0.180
Advising on the benefits of COVID-19 vaccination	4 (1)	4 (1)	0.059
Offering help and support regarding COVID-19 vaccination	3 (2)	4 (2)	0.496
Addressing common worries and misconceptions	4 (2)	4 (2)	0.453
Counseling patients who indicate they are uncertain or do not intend to be vaccinated	4 (2)	4 (1)	1
Motivation^b^			
It is important to intervene with patients/communities in order to reduce COVID-19 vaccination hesitancy.	4 (1)	5 (1)	0.083
It is important to support COVID-19 vaccination in high-risk and socially deprived populations.	5 (1)	5 (0)	1
I will intervene with COVID-19 vaccination only with high-risk patient with serious comorbidities.	4 (3)	3.5 (3)	0.671
Raising the issue of vaccination will create a problem in my professional relationship with patients.	2 (1)	2 (1)	0.180
Opportunity^b^			
It is the government’s, not the healthcare professional’s role to address misconceptions over COVID-19 vaccination.	3 (2)	1 (3)	**0.041**
I cannot assure my patients regarding the safety of COVID-19 vaccines.	3 (2)	2.5 (2)	0.480
Intentions^c^ I intend to…			
Ask all my patients whether they have been vaccinated against COVID-19	4 (1)	4 (1)	1
Inform all my patients about their COVID-19 vaccination options	4 (1)	4.5 (2)	1
Address COVID-19 vaccination hesitancy with all my patients	4 (1)	4 (1)	0.157
Offer brief COVID-19 vaccination hesitancy interventions to all my patients who are unsure or unwilling to be vaccinated	4 (1)	4 (1)	1

IQR, interquartile range.

^a^Assessment question: On a scale of 1 to 5, how strongly would you agree with the following statements. I am confident in… (Response options (1) strongly disagree through to (5) strongly agree).

^b^Assessment question: On a scale of 1 to 5, how strongly would you agree with the following statements. (Response options (1) strongly disagree through to (5) strongly agree).

^c^Assessment question: On a scale of 1 to 5, how strongly would you agree with the following statements. I intend to…. (Response options (1) strongly disagree through to (5) strongly agree).

^d^Wilcoxon Signed Rank test value of *p* calculated based on sample of providers for which data was available at both timepoints being compared.The bolded values represent those with statistical significance.

Positive changes documented in several constructs relating to provider attitudes, beliefs and motivated related to COVID-19 (Table 4). There was a significant change in providers attitudes regarding the role of healthcare professionals versus government in addressing COVID-19 vaccine hesitancy among residents (*p* = 0.041).

Ninety percent of providers strongly agreed or agreed that they planned to use the information and skills provided by the training in their clinical practice (Table 4). No further significant changes were documented in practice specific intentions.

## Discussion

### Main findings

This eLearning CME was designed to support health care professionals with having effective conversations with patients about COVID-19 vaccination. The training supports health care professionals with how to raise the discussion with patients and provide VBA on COVID-19 vaccination. It also addresses how MI techniques can be used to guide discussions with patient who are ambivalent or hesitant about COVID-19 vaccination. The training was informed by recent experience with addressing vaccine hesitancy internationally and we attempted to tailor the skills training to the local Greek dialog and context.

The pilot evaluation indicated high levels of satisfaction among providers and positive but mixed effects on providers confidence, attitudes, and intentions. The present study reported on a small pilot evaluation and a larger study would be useful for further examining the pre-post intervention effects.

We adapted existing evidence-based behavior change and counseling techniques with proven efficacy in changing other behaviors (Very Brief Advice) and uncertainty or resistance about behavior change (motivational interviewing) to address COVID-19 vaccine hesitancy. Since the initiation of this project several groups have examined the role of MI in addressing (22–25). This project adds to international work regarding the adaptation of MI counseling techniques for COVOD-19 vaccine hesitancy that has been tailored to the national context in Greece.

### Implications for research and practice

The project mobilized existing knowledge and expertise to rapidly develop and deploy the educational intervention to rollout in parallel to the planned population-wide vaccination of residents in Greece. The assets created as part of the present project, including the eLearning and outreach supports are hosted on the Primary Care Training Hub of the University of Crete. The interventions strategies used for addressing COVID-19 vaccine hesitancy may have broader learnings for addressing vaccine hesitancy for other immunization programs (e.g., influenza). Future studies could be developed in other countries and involve other professionals (e.g., speech therapists, nurses, physical therapists). There will be an expected need to update the training to address booster doses of COVID-19 vaccine hesitancy that are tailored to residents’ beliefs about the risk and value of booster doses. Future research should seek to incorporate novel eLearning tools such as massive open online courses (MOOCs) (26, 27). MOOCs are designed to promote quick and effective continuous education which are designed to reach large numbers of learners and make use of open access policies (27). MOOCs which are continuously updated are particularly relevant to subject areas such as COVID-19 vaccine hesitancy which is rapidly evolving and requires regular updates to ensure content remains relevant, evidence-based and addressing priorities over time (e.g., vaccine booster doses). While in the present eLearning was rated strongly in terms of learner satisfaction. Future research should also seek to ensure digital competencies of learners are considered in design process to enhance learner experience and participation rates (28). For example, reducing course length or organizing course content into “core” and “optional” may have served to assist with increasing course completion rates among participants.

This pilot study had limitations. Firstly, the sample size was limited and loss to follow-up fairly large. At the time of this study there were no published tools which adapt assessment of capability, motivation, opportunity and intentions for COVID-19 vaccination and vaccine hesitancy. Our team developed a customized tool for use in this pilot study. We adapted existing evaluation tools for the assessment of training programs to assess COMB-B constructs as it relates to COVID-19 vaccine hesitancy practice behaviors. Further research in the field to validate and refine tools for this purpose would be recommended. It would be relevant for future research to examine the predictive value of these constructs as well as provider socio-demographic characteristics including personal vaccine status of providers on practice behaviors.

## Conclusion

This project has resulted in new training assets for use by health and social professional tailored to the nationally context in Greece which can now be used for dissemination nationally to support vaccination uptake.

## Data availability statement

The raw data supporting the conclusions of this article will be made available by the authors, without undue reservation.

## Ethics statement

The studies involving humans were approved by University of Crete Research Ethics Committee. The studies were conducted in accordance with the local legislation and institutional requirements. The participants provided their written informed consent to participate in this study.

## Author contributions

SP: Conceptualization, Formal analysis, Funding acquisition, Investigation, Methodology, Writing – original draft, Writing – review & editing. MA: Conceptualization, Data curation, Funding acquisition, Investigation, Methodology, Project administration, Writing – original draft, Writing – review & editing, Formal analysis. MG: Data curation, Formal analysis, Investigation, Project administration, Writing – review & editing. XP: Project administration, Writing – review & editing, Methodology, Resources, Supervision, Visualization. EA: Methodology, Visualization, Writing – review & editing, Investigation. CL: Investigation, Methodology, Writing – review & editing, Conceptualization, Data curation, Funding acquisition, Project administration, Writing – original draft.

## Footnotes

^1^ https://apps.who.int/iris/bitstream/handle/10665/340751/WHO-EURO-2021-2281-42036-57837-eng.pdf
